# Clustering 1-dimensional periodic network using betweenness centrality

**DOI:** 10.1186/s40649-016-0031-1

**Published:** 2016-10-21

**Authors:** Norie Fu, Vorapong Suppakitpaisarn

**Affiliations:** 1 JST, ERATO Kawarabayashi Large Graph Project, Global Research Center for Big Data Mathematics, National Institute of Informatics (NII), 2-1-2 Hitotsubashi, Chiyoda-ku, Tokyo, 101-0003 Japan; 2 Department of Computer Science, The University of Tokyo, 7-3-1 Hongo, Bunkyo-ku, Tokyo, 113-0033 Japan

**Keywords:** Efficient algorithms for social computing, Clustering algorithm, Social influence, Opportunistic network, Periodic graph

## Abstract

**Background:**

While the temporal networks have a wide range of applications such as opportunistic communication, there are not many clustering algorithms specifically proposed for them.

**Methods:**

Based on betweenness centrality for periodic graphs, we give a clustering pseudo-polynomial time algorithm for temporal networks, in which the transit value is always positive and the least common multiple of all transit values is bounded.

**Results:**

Our experimental results show that the centrality of networks with 125 nodes and 455 edges can be efficiently computed in 3.2 s. Not only the clustering results using the infinite betweenness centrality for this kind of networks are better, but also the nodes with biggest influences are more precisely detected when the betweenness centrality is computed over the periodic graph.

**Conclusion:**

The algorithm provides a better result for temporal social networks with an acceptable running time.

## Background

**Fig. 1:**
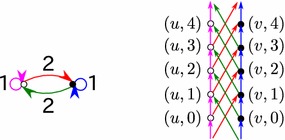
A 1-dimensional periodic graph (*right*) and its associate static graph (*left*)

In this paper^1^, we propose a clustering method for temporal networks specified by 1-dimensional periodic graphs. A *k*-dimensional periodic graphs is a graph constructed by placing a finite graph to all cells in a *k*-dimensional lattice. That finite graph is called static graph. A 1-dimensional periodic graph is then a graph that has an infinite copies of the static graph. Each of the copies is placed on an integer of a number line. An example of a 1-dimensional periodic graph, together with its static graph, can be found in Fig. 1.

The 1-dimensional periodic graphs have a wide range of applications. These include the model that illustrated how people move in specific situations, as proposed by Sekimoto et al. [2], and the model that was used for finding the optimal train schedule based on train demand, as proposed by Orlin, Serafini, and Ukovich [3–5]. In this paper, we will focus on the application of the graphs to opportunistic communication where each object in sensor networks communicates with the others in every given period of time [6].

Each sensor *i* in the opportunistic networks is represented by a node *i* in a static graph. In a periodic graph generated by the static graph, there are infinite copies of the node *i*, $(i, \langle h \rangle )$ for $h \in \mathbb {Z}$. A node $(i, \langle h \rangle )$ in the periodic graph represents the sensor *i* for time *h*. If the communication time between the sensor *i* and a sensor *j* is *r*, we know that the information from *i* at time *h*, represented by the node $(i, \langle h \rangle )$ in the periodic graph, reaches *j* at time $h + r$. Since the sensor *j* at time $h + r$ is represented by a node $(j, \langle h + r \rangle )$, we link the node $(i, \langle h \rangle )$ with the node $(j, \langle h + r \rangle )$. Similarly, for any communication between two sensors $i'$ and $j'$ that takes $r'$ period of time, we link $(i', \langle h'\rangle )$ with $(j', \langle h' + r' \rangle )$ for all $h' \in \mathbb {Z}$. Thus, we get the periodic structure as shown in Fig. 1.

Because of the applications discussed in the previous paragraph, there are many works that proposed algorithms for the graph. Those works include a work by Orlin, who proposes algorithms to determine weakly connected components [7], strongly connected components [7], Eulerian paths [7], minimum cost spanning trees [7], maximum flows [8], and minimum cost flows [9]. Later, Cohen and Megiddo propose algorithms to test bipartiteness [10] and detect cycles [11] in the periodic graphs. Besides that, an algorithm to test a planarity of a given periodic graph is proposed by Iwano and Steiglitz in [12], and an algorithm to find a shortest path for an arbitrary periodic graph is proposed by Höfting and Wanke in [13]. In [14], Fu proposes the shortest path algorithm for a special class of planar periodic graphs. The result shows that planarity can help in speeding up the computation of the previous shortest path algorithm.

Although there are many periodic extensions for many basic algorithmic problems in the literature, there are not many data mining or machine learning techniques specifically proposed for them. In this paper, we will focus on clustering problem, one of the most common problems in data mining. We consider a clustering method based on betweenness centrality.

Centrality is a notion that defines the importance of nodes and edges in a given graph [15]. It is discussed in [16] that, if we remove all edges with high centrality from the graph, each connected component in the remaining graph can represent a cluster. Among all centrality indexes, betweenness centrality is known as one of the most common indexes [17]. Besides its application in clustering, we can also use the betweenness value to measure the influence of each node in the network [17, 18].

It is also possible to cluster a periodic graph using the betweenness centrality of its static graph. However, we strongly believe that the clustering method ignores some important information that we have in the periodic graph. A method which considers more information should help us find better clustering results.

### Our contribution

The definition of betweenness centrality in the previous works is made only for finite graphs. As the number of nodes of an periodic graph is infinite, it is not clear if we can directly use the definition in our setting. In [19], we extend the definition to the periodic case that preserve the meaning of the betweenness centrality. Besides that, we give a mathematical proof to show that the new definition is valid, using theoretical results on integer programming. Also, we give an algorithm to compute this betweenness centrality for a given network based on dynamic programming and recurrence relations. The algorithm is demonstrated to run in polynomial time when the input graph is VAP-planar, i.e., there exists a drawing of the input periodic graph with no line crossing and no finite area containing an infinite number of nodes.

Although VAP-planar periodic graphs are used in many practical applications, graphs obtained from opportunistic communication are usually not VAP-planar graphs. This motivates us to consider a different class of periodic graphs in this paper. In this class, we assume that the graphs have the following properties:

1. Recall that a periodic graph contains a repetitive structure, and each part corresponds to a snapshot at time *h*. Let $V_h := \{(i,\langle h' \rangle ) : h' = h\}$ be a set of nodes in time *h*, and the transit value of an edge from $V_h$ to $V_{h^*}$ be $h^* - h$. We require that the transit values of all edges are positive.
2. We require that the edge weights, which are used for deciding which paths are the shortest paths, are equal to their transit value.
3. We require that the least common multiples of all transit values are bounded by a constant *K*.

The transit value corresponds to the communication time between two nodes in our model. Clearly, the communication time must be positive. Also, since the shortest paths should be the communication paths that take smallest amount of time, it is natural to consider the weight of each edge as the communication time between nodes. Thus, we strongly believe that the second assumption is also natural.

The least realistic requirement could be the third one. However, we observe that there are not usually many distinct values of communication time in most of the real-world datasets. Therefore, the least common multiplier of the transit values is usually small.

In ‘Algorithm’ section, we propose an algorithm that can find a betweenness centrality of a periodic graph satisfying those three conditions. The ideas behind the algorithm are dynamic programming and recurrence relations. The asymptotic complexity of our algorithm is $O(|\mathcal {V}|^3)$ when $|\mathcal {V}|$ is the number of nodes in the input static graph.

Since there is no algorithm for clustering the periodic graph proposed in literature, we compare our computation with the computation time of the fastest algorithm for finite graphs [20], for which the asymptotic complexity is $O(|\mathcal {V}||\mathcal {E}|)$ when $|\mathcal {V}|$ is the number of nodes and $|\mathcal {E}|$ is the number of edges in the input graph. Although our asymptotic computation time is larger than the previous algorithm, the experimental results in ‘experimental results’ section show that the computation time for both is not that different in practice.

Our algorithm takes 3.2 s for an opportunistic network with 125 nodes and 455 edges constructed from the data according to Fournet and Barrat [21]. By means of our betweenness centrality on the periodic graph, we can find clusters with around 50 % higher precision and recall, compared to the results obtained from applying the classical definition to its static graph. Besides that, we can spread information to 3–10 % more nodes in periodic graph, if we use nodes with higher periodic betweenness instead of nodes with higher betweenness in static graph.

### Related works

It is important to note that the 1-dimensional periodic graphs in this paper are different from the 1-dimensional graphs in papers on complex networks such as [22, 23]. While each integer on a number line has exactly one node in the 1-dimensional graph, each integer has a whole static graph, which can have more than one node, in the 1-dimensional periodic graph.

There are many works studying properties that are changed or updated over time (see [24, 25] for reviews on this topic). There are also works which use those properties to propose an algorithm for clustering, such as Aynaud et al. [26] and influence maximization Ohsaka et al. [27]. Besides, when the input graph slightly changes, there exist works that can efficiently update the value of the influence maximization, e.g., Ohsaka et al. [28] and betweenness centrality, e.g., by Hayashi et al. [29].

Besides the opportunistic networks previously discussed, communication networks in which nodes send an information to others at every given periods are also studied by other models (e.g., models proposed in [30]). In [31, 32], Lahiri and Berger-Wolf model the communication by a set of subgraphs of social networks. Each subgraph contains a communication at a specific time. The authors devise an algorithm that can detect all subgraphs in polynomial time in the paper. The centrality of nodes in this model is discussed by Pfeiffer, and Neville in [33]. Although the model is used in a wide range of applications such as biological studies [34, 35], it assumes that information is immediately arrived to its receiver after the transmission. That makes the model slightly different from ours.

The dynamic in communication periods between two nodes is also a well-studied research topic. While, in many studies (e.g., [36, 37]), the distribution of periods between two communications is approximated by Poisson processes, the study by Barabasi [38] shows that the human communications consists of several burst periods and there is a long duration between each of the periods. In this work, we consider the case when the long duration has a periodic pattern.

While we select the most important nodes to maximize the influence in this paper, there are also works that select a transition probability to maximize the delivery efficiency of a given network (e.g., [39–41]).

Also, there are works that transform pseudoperiodic time series to a complex network, and use properties of the network to analyze the series (e.g., [42, 43]). Although the graph in those works are obtained from dynamic series, the graph is static. Hence, methods used for analyzing their graphs are different from ours.

## Problem definition

In ‘Definition of Periodic Graphs’ subsection, we will give a formal definition of periodic graphs. Then, in ‘Periodic Betweenness Centrality’ subsection, we will give a definition of the betweenness centrality for the periodic graphs. The betweenness definition has been introduced in our previous work [19].

### Definition of periodic graphs

A *periodic graph* is an infinite repetition of a finite structure. We call that finite structure as *static graph*, and define it in Definition 1. Then, we define the periodic graph in Definition 2.

#### **Definition 1**

(*Static Graph*) The tuple $\mathcal {G} = (\mathcal {V}, \mathcal {E}, \mathrm {w})$ of a vertex set $\mathcal {V} = \{1, \dots , n\}$, a set of directed edges with vector labels $\mathcal {E} = \{e^{(1)}, \dots , e^{(m)}\} \subseteq (\mathcal {V} \times \mathcal {V}) \times \mathbb {Z}^d$, and a weight function $\mathrm {w}: \mathcal {E} \rightarrow \mathbb {R}_{> 0}$ is called a **static graph**.

#### **Definition 2**

(*Periodic Graph*) For a static graph $\mathcal {G} = (\mathcal {V}, \mathcal {E}, \mathrm {w})$, the **periodic graph** $G = (V, E, \hat{\mathrm {w}})$ **generated by** $\mathcal {G}$ is an infinite graph with weights of edges, such that $V =\mathcal {V} \times \mathbb {Z}^d$,

$$\begin{aligned} E = \{((i, \mathbf {h}), (j, \mathbf {h} + \mathbf {g})) : \mathbf {h} \in \mathbb {Z}^d, ((i, j), \mathbf {g}) \in \mathcal {E}\} \subset V \times V, \end{aligned}$$

and

$$\begin{aligned} \hat{\mathrm {w}}: E \rightarrow \mathbb {R}_{> 0},~\hat{\mathrm {w}}((i, \mathbf {h}), (j, \mathbf {h} + \mathbf {g})) = \mathrm {w}(((i, j), \mathbf {g})). \end{aligned}$$

If $\mathcal {G}$ has *d*-dimensional transit vectors, then we call *G* a *d* *-dimensional periodic graph*. Unless otherwise specified, we use $\mathcal {G} = (\mathcal {V}, \mathcal {E}, \mathrm {w})$ to denote a static graph, and $G = (V, E, \hat{\mathrm {w}})$ to denote the periodic graph generated by $\mathcal {G}$. In this paper, we consider only the case when $d = 1$, which is a case when the vector $\mathbf {h}$ and $\mathbf {g}$ in the previous definition are 1-dimensional vectors of integer. For simplicity, we will assume that both the static graphs and the periodic graphs considered in this paper are weakly connected. If the input graph is not weakly connected, the betweenness results of the graph can be straightforwardly obtained from the betweenness results of its connected components.

#### **Definition 3**

(*Length of a walk*) Given a walk *W* with edges *F* on *G*, we define the *length of* *W* as $\sum \limits _{e \in F} \hat{\mathrm {w}}(e)$. Analogously on $\mathcal {G}$, we define the **length of a walk** $\mathcal {W}$ with edges $\mathcal {F}$ as $\sum \limits _{e \in \mathcal {F}} \mathrm {w}(e)$.

The *distance from* *s* *to* *t* in *G*, denoted by $d_{G}(s,t)$ (or simply *d*(*s*, *t*) if the graph is omissible), is the length of a walk from *s* to *t* in *G* such that its length is minimized. This kind of walk is also known as a *shortest path*.

### Periodic betweenness centrality

Let $H = (U, F)$ be an undirected graph. For any two vertices $s, t \in U$, we denote by $\sigma _{s,t}^{H}$ the number of distinct shortest paths between *s* and *t* in *H*, and $\sigma _{s,t}^{H}(v)$ the ones that contains *v*.

The *betweenness centrality of a vertex* *v* *on a finite graph* $H = (U, F)$ is defined as

$$\begin{aligned} g^H(v) = \sum \limits _{s \ne v \ne t} \frac{\sigma _{s,t}^H(v)}{\sigma _{s,t}^H}. \end{aligned}$$

We will abbreviate $g^H(v)$, $\sigma _{s,t}^{H}$, $\sigma _{s,t}^{H}(v)$ as *g*(*v*), $\sigma _{s,t}(v)$ and $\sigma _{s,t}$, when the graph is obvious from its context.

Now, let $G = (V, E)$ be a graph that could be infinite, and fix the vertex $\nu$ betweenness centrality of which is to be computed. We denote the set $V_D(\mu )$ as follows:

$$\begin{aligned} V_{D}(\mu ) := \left\{ \omega \in V: d_G(\mu , \omega )< D\right\} \cup \left\{ \omega \in V: d_G(\omega , \mu ) < D\right\} \end{aligned}$$

where $D \in \mathbb {R}_{\ge 0}$. Intuitively, the set $V_D(\mu )$ is a set of vertices to which the distance from $\mu$ is no longer than *D*, or from which the distance to $\mu$ is no longer than *D*. Unless otherwise specified, we abbreviate $V_D(\mu )$ by $V_D$ when $\mu = \nu$. Let $G_D$ the subgraph of *G* induced by a set of nodes $V_D$. Our betweenness centrality of a node $\nu \in V$, $pbc(\nu )$, can be defined as follows.

#### **Definition 4**

(*Periodic Betweenness Centrality*) For $\nu \in V$, the **periodic betweenness centrality of** $\nu$ **on** *G* is

$$\begin{aligned} pbc(\nu ) = \lim _{D \rightarrow +\infty } \frac{g^{G_D}(\nu )}{|V_D|^2}. \end{aligned}$$

We note that the periodic betweenness centrality is an extension of the betweenness centrality on a finite graph $H = (U, F)$. It is straightforward to show that the periodic betweenness centrality of any $u \in U$, *pbc*(*u*), is equal to $g(u) / |U|^2$, i.e., we can calculate the value of *pbc*(*u*) by dividing the betweenness centralities of all nodes by $|U|^2$. Since the main purpose of the betweenness is to compare the centrality of the vertices, scaling does not affect the result.

In [19], we show that, for all 1-dimensional periodic graph *G* and all nodes $\nu$ of *G*, the value of $pbc(\nu )$ always converges to some positive real number. In the same paper, we give an algorithm that can output $pbc(\nu )$ in polynomial time, if the given periodic graph is VAP-planar.

## Algorithm

We do not give an algorithm for a general 1-dimensional one periodic graph, but a periodic graph used for capturing behaviors of an opportunistic network. Therefore, we assume that the input periodic graphs must satisfy some assumptions given in the ‘Assumption’ subsection.

In the ‘Dynamic Programming Idea’ subsection, we will give an algorithm for finding a betweenness centrality for nodes in *finite* graphs. The algorithm does not improve the state-of-the-art algorithm for the finite graphs, but it can be extended to an algorithm for the periodic graph. We give the extended algorithm in the ‘Recurrence Relations’ subsection, and prove some of its properties in the ‘Properties of $S_v$’ subsection.

### Assumption

Before formally defining our assumption, we give the following definitions.

#### **Definition 5**

(*Positive Periodic Graph*) Let $\mathcal {G} = (\mathcal {V}, \mathcal {E}, \mathrm {w})$ be a static graph of a 1-dimensional periodic graph *G*. If, for all $e^{(t)} = (i, j, \langle g \rangle ) \in \mathcal {E}$, the value of *g* is positive, then *G* is a **positive periodic graph**.

Since a cycle $\langle (i_1, i_2, \langle g_1 \rangle ), (i_2, i_3, \langle g_2 \rangle ), \dots , (i_m, i_1, \langle g_m \rangle ) \rangle$ must have $\sum _i g_i = 0$, and the summation of the values of $g_i$ for all paths in a positive periodic graph is positive. We know that a positive periodic graph does not contain a cycle.

#### **Definition 6**

(*Weight-Transit Periodic Graph*) Let $\mathcal {G} = (\mathcal {V}, \mathcal {E}, \mathrm {w})$ be a static graph of a 1-dimensional periodic graph *G*. If, for all $e^{(t)} = (i^{(t)}, j^{(t)}, \langle g^{(t)} \rangle ) \in \mathcal {E}$, $\mathrm {w}\left( e^{(t)}\right) = g^{(t)}$, then *G* is a **weight-transit periodic graph**.

Also, we define a period of a periodic graph *G* as follows:

#### **Definition 7**

(*Period of Periodic Graph*) Let $\mathcal {G} = (\mathcal {V}, \mathcal {E}, \mathrm {w})$ be a static graph of a 1-dimensional periodic graph *G*. Let $\mathcal {E} = \{e^{(1)}, \dots , e^{(|\mathcal {E}|)}\}$ and, for all $e^{(t)}$, $e^{(t)} = (i^{(t)}, j^{(t)}, \langle g^{(t)} \rangle )$. The **period** of *G*, *p*(*G*), is defined as

$$\begin{aligned} p(G) = \mathrm {lcm}\left( g^{(1)}, \cdots , g^{(|\mathcal {E}|)}\right) . \end{aligned}$$

We assume the our input periodic graph *G* (or $\mathcal {G}$) must satisfy the following conditions:

1. *G* is a positive periodic graph, and
2. *G* is a weight-transit periodic graph.

When we model an opportunistic network using a periodic graph, each node in the static graph, $i \in \mathcal {V}$ represents a person or a sensor node. A node $(i,t) \in V$ represents a person *i* at a time slot *t*. An edge from (*i*, *t*) to $(j,t')$ represents a communication which begins at *i* at time *t* and arrives at *j* at time $t'$. Therefore, $g := t' - t$ represents a communication time from *i* to *j* in our model. Because it is natural to assume that the communication time is positive, we believe that the first assumption is natural. Also, it is natural to assume that the weights of edges are equal to the communication time. Furthermore, it is natural to assume that our input graph is a weight-transit periodic graph.

Since the computation time of our algorithm is bounded by a polynomial function of $|\mathcal {V}|$, $|\mathcal {E}|$, and *p*(*G*), our algorithm will take a long time to terminate if the period of the graph *G* is large. Thus, we also assume that the period is much smaller than $|\mathcal {V}|$ and $|\mathcal {E}|$. The value of the least common multiple is actually very small in our datasets, since the number of distinct values of *g* in an opportunistic network is usually not greater than 5.

### Dynamic programming idea

Recall the notation $G_{D}$ defined in the previous section. For any $g' \in \mathbb {Z}$, we denote

$$\begin{aligned} V^{\left( g'\right) } := \{(i,\langle g \rangle ) \in V : g = g'\}. \end{aligned}$$

Assume without loss of generality that $\nu \in V^{(0)}$. We know that the number of shortest paths $\sigma _{s,t}^{G_{D}}(\nu ) > 0$, only if $s \in V^{(\ell )} \cup \{\nu \}$ and $t \in V^{(k)} \cup \{\nu \}$ for some $\ell < 0$ and $k > 0$. Otherwise, $\sigma _{s,t}^{G_{D}}(\nu ) = 0$.

In this subsection, we will give an idea about how we calculate the value $\sigma _{s,t}^{G_{D}}(\nu ) > 0$ for some specific $D \in \mathbb {Z}_+$, $s \in V^{(\ell )}$ and $t \in V^{(u)}$. To calculate the above value, we will first compute the number of the shortest paths from *s* to $\nu$, denoted by $\sigma _{s,\nu }$, and the distance from *s* to $\nu$, denoted by $d(s, \nu )$, in $G_{D}$. Our ideas behind the computation of those values are shown in Algorithm 1.

To find the number of the shortest paths from *s* to *t*, in Algorithm 1, $\sigma _{s,t}$, we calculate the number of the shortest paths from *s* to all nodes in $V^{(\ell + 1)}, V^{(\ell + 2)}, \dots , V^{\ell '}$ when $\ell '$ is an integer such that $t \in V^{\ell '}$. Recall our assumption that, for all edges (*u*, *v*) such that $u \in V^{(i)}$ and $v \in V^{(j)}$, we have $j > i$. We know that all paths to a node *v* in $V^{(i)}$ must pass through a node in $V^{(i')}$ for $i' < i$ before arriving at *v*. Based on this idea, we calculate the number of shortest paths and the distances to all nodes in $\bigcup \nolimits _{\ell< i' < i}V^{(i')}$, and use that information to calculate the values for $V^{(i)}$. The set $S_v$ obtained from the function $\arg \min$ in Line 7 denotes a set of all edges (*u*, *v*) that minimize the value $d(s,u) + \mathrm {w}\left( (u,v)\right)$. Since *d*(*s*, *u*) denotes the shortest distance from *s* to *u* and $\mathbf {w}((u,v))$ denotes the distance from *u* to *v*, we know that $S_v$ denotes all incoming edges to *v* that is a part of a shortest path between *s* and *v*. Thus, the number of shortest paths from *s* to *v* is the summation of the number from *s* to members of $S_v$, as described in Line 8.
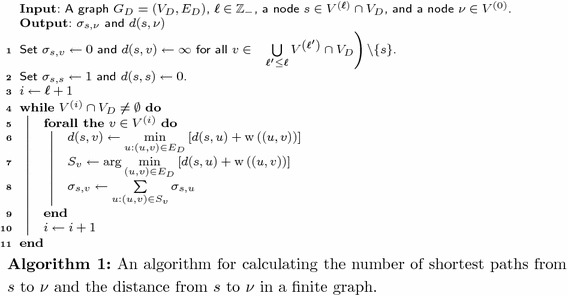


Algorithm 1 is clearly slower than the fastest algorithm for the betweenness calculations proposed in [20]. However, the idea used in the algorithm can be extended to an infinite periodic graph in the following subsection. We will show the correctness and the computation time of the algorithm in the following theorem.

#### **Theorem 1**

*Algorithm 1 can calculate the values of* $\sigma _{s,\nu }$ *and* $d(s,\nu )$ *in* $O(|E_D|)$ *when* $E_D$ *is the set of edges in* $V_D.$

#### Proof

The bottleneck of Algorithm 1 is encountered in Lines 6–8 of the algorithm. Because each edge will be considered only once in those lines, the computation time of the algorithm is $O(|E_D|)$.

We will prove the correctness of the algorithm by induction on the variable *i*. It is clear that there are no paths from *s* to *v* when the node *v* is in $\left( \bigcup \nolimits _{\ell ' \le \ell } V^{\left( \ell '\right) } \cap V_{D} \right) \backslash \{s\}$, because our periodic graph is positive. Thus, $\sigma _{s,v} = 0$ and $d(s,v) = \infty$, as assigned in Line 1. Because our positive periodic graph contains no cycle, it is clear that the only path from *s* to *s* is an empty set. Therefore, $\sigma _{s,s} = 1$ and $d(s,s) = 0$, as shown in Line 2.

Consider a node $v \in \bigcup \nolimits _{\ell ' < i} V^{\left( \ell '\right) } \cap V_D$. Assume that Algorithm 1 can give correct values of $\sigma _{s,v}$ and *d*(*s*, *v*). Since $v \ne s$, we know that a node *v* needs at least one edge to reach the node *s*. Therefore, *d*(*s*, *v*) can be calculated as shown in Line 6 of the algorithm. All the shortest paths to the node *v* are the paths to some other nodes *u* added with an edge from *u* to *v*. The number of the shortest paths is the summation of the number of shortest paths to each neighbor *u* of *v* such that $d(s,u) + \mathrm {w}((u,v))$ is minimized, as calculated in Lines 7–8. $\square$

Using the same method, we can calculate $\sigma _{s,t}$ and *d*(*s*, *t*) for all $s \in \bigcup \limits _{\ell ' < 0} V^{(\ell ')}$ and $t \in \bigcup \nolimits _{\ell ' > 0} V^{(\ell ')}$. Also, by inverting the sides of all edges, we can calculate the values of $\sigma _{\nu ,t}$ and $d(\nu , t)$ for each $t \in \bigcup \nolimits _{\ell > 0 } V^{(\ell )}$. Based on these values, we know that there exist some shortest paths from *s*, *t* that pass through $\nu$, only if $d(s,t) = d(s,\nu ) + d(\nu , t)$. If there are some shortest paths, it is clear that the number $\sigma ^{G_D}_{s,t}(\nu )$ is equal to $\sigma ^{G_D}_{s,\nu } \cdot \sigma ^{G_D}_{\nu ,t}$.

In short, we can calculate the betweenness centrality of $\nu$ by

$$\begin{aligned} g^{G_D}(\nu ) = \sum _{s \ne \nu \ne t} p_{s,t,\nu }\frac{\sigma ^{G_D}_{s,t}(\nu )}{\sigma ^{G_D}_{s,t}} = \sum _{s \ne \nu \ne t} p_{s,t,\nu }\frac{\sigma ^{G_D}_{s,\nu } \cdot \sigma ^{G_D}_{\nu ,t}}{ \sigma ^{G_D}_{s,t}}, \end{aligned}$$

where $p_{s,t,\nu } = 1$ if $d(s,t) = d(s,\nu ) + d(\nu ,t)$, and $p_{s,t,\nu } = 0$ otherwise.

### Recurrence relations

Recall that $V^{(\ell )}$ can be written in the form: $\{(0, \langle \ell \rangle ), \dots , (n, \langle \ell \rangle )\}$. When $D \rightarrow \infty$ and Algorithm 1 do not terminate, we will find a betweenness centrality by solving a recurrence relation for $\sigma _{s,\nu }$, $\sigma _{\nu ,t}$, $\sigma _{s,t}$ and $|V_D|^2$.

Let $g_\mathrm{max} := \max \{g : ( i,j, \langle g \rangle ) \in \mathcal {E} \}$. Based on Algorithm 1, we know that $\sigma _{s,(v,\langle i \rangle )}$ can be written in the form:

$$\begin{aligned} \sigma _{s,(v,\langle i \rangle )} = \sum _{r = 1}^{g_\mathrm{max}} \sum _{(v', \langle i - r \rangle ) \in V^{(i - r)}} c_{i,r,v,v'} \sigma _{s,(v',\langle i-r \rangle )}. \end{aligned}$$

$c_{i,r,v,v'} = 1$ when $\left( (v',\langle i - r \rangle ), (v,\langle i \rangle ) \right) \in S_{(v,\langle i \rangle )}$, and $c_{i,r,v,v'} = 0$ otherwise.

Let $s \in V^{(\ell )}$ for some $\ell \in \mathbb {Z}_-$. When the given periodic graph is weight-transit, we will argue in the next subsection that, for all $i > \ell$, $c_{i,r,v,v'} = 1$ if $((v', \langle i - r \rangle ), (v,\langle i \rangle )) \in E$ and $(v', \langle i - r \rangle )$ are reachable from *s*. Otherwise, $c_{i,r,v,v'} = 0$. Since $\sigma _{s, (v', \langle i - r \rangle )} = 0$ for any node $(v', \langle i - r \rangle )$ is unreachable from *s*, we can still get the same solution even when we set $c_{i,r,v,v'}$ to 1. We can set $c_{i,r,v,v'} = 1$ if $((v', \langle i - r \rangle ), (v,\langle i \rangle )) \in E$, and $c_{i,r,v,v'} = 0$ otherwise. From the periodicity of our graph, we have $c_{i,r,v,v'} = c_{j,r,v,v'}$ for any $i,j \in \mathbb {Z}$. We can simplify the notation $c_{i,r,v,v'}$ to $c_{r,v,v'}$, and get the following recurrence relation:

$$\begin{aligned} \sigma _{s,(v,\langle i \rangle )} = \sum _{r = 1}^{g_\mathrm{max}} \sum _{(v', \langle i - r \rangle ) \in V^{(i - r)}} c_{r,v,v'} \sigma _{s,(v',\langle i-r \rangle )}. \end{aligned}$$

Since we can calculate the value of $\sigma _{s, (v,\langle i \rangle )}$ for all $v \in V$ using Algorithm 1, we can solve the recurrence relation to find a closed form for $\sigma _{s,(v,\langle i \rangle )}$.

The number of the shortest paths from $(v',\langle i' \rangle )$ to $(v,\langle i \rangle )$ is equal to the number of the shortest paths from $(v', \langle i'-i \rangle )$ to $(v,\langle 0 \rangle )$, since the transition on a periodic graph does not change the number of paths. Hence, $\sigma _{(v', \langle i' \rangle ), (v,\langle i \rangle )} = \sigma _{(v',\langle i'-i \rangle ), (v,\langle 0 \rangle )}$. Let $s = (v',\langle i' \rangle )$. As we have the value of $\sigma _{(v',\langle i' \rangle ), (v,\langle i \rangle )}$ for all $v', v, i$ from the calculation in the previous paragraph, we can use those results to get $\sigma _{(v',\langle j \rangle ), (v,\langle 0 \rangle )}$ for all $v',v,j$. When $\nu = (v,\langle 0 \rangle )$, we can have the closed form for the number of the shortest paths from all nodes to $\nu$.

Using the similar idea, we can find closed forms for $\sigma _{(v,\langle i \rangle ),t}$, $\sigma _{s,t}$, and $|V_D|^2$. From these closed forms, we can calculate $pbc(\nu )$ defined in ‘Problem Definition’ section.

#### **Theorem 2**

*Using our method, we can calculate* $pbc(\nu )$ *for all* $\nu \in \mathcal {V}$ *in* $O\left( |\mathcal {V}|^3p^3(G)\right)$ *where* $p(G) := \mathrm {lcm}\left( \{g : (i,j, \langle g) \in \mathcal {E} \} \right)$.

#### Proof

By means of the properties of $S_v$ shown in the following subsection, we can obtain recurrence relations for $\sigma _{s,(v,\langle i \rangle )}$, $\sigma _{(v,\langle i \rangle ),t}$, $\sigma _{s,t}$, and $|V_D|^2$ in $O(|\mathcal {E}|p(G))$. In this paper, we solve the recurrence relations by eigenvalue decomposition. Since the number of variables in our recurrence relations is $|\mathcal {V}|p(G)$, the size of a square matrix constructed from the recurrence relation is $|\mathcal {V}|p(G)$. The computation time for the eigenvalue decomposition of a square matrix size *s* is $O(s^3)$. Thus, the computation time used for solving the recurrence relations is $O\left( |\mathcal {V}|^3p^3(G)\right).$

From the previous paragraph, we know that the computation time of our method is $O\left( |\mathcal {E}|p(G) + |\mathcal {V}|^3p^3(G)\right) = O\left( |\mathcal {V}|^3p^3(G)\right)$. $\square$

From the previous theorem, we know that the running time of our algorithm is polynomial of $|\mathcal {V}|$, $|\mathcal {E}|$, and *p*(*G*). Since we assume that *p*(*G*) is smaller than a constant *K*, our computation time, $O(|\mathcal {V}|^3)$, is slightly larger than those of the fastest algorithm for static graphs, which is $O( |\mathcal {V}||\mathcal {E}|)$ for a static graph with $|\mathcal {V}|$ nodes and $|\mathcal {E}|$ edges [20].

### Properties of $S_v$

In this subsection, we will prove a property of the set $S_v$ defined in Algorithm 1. Let $\mathbf {t}$ be a function from a path $\langle e_1, \dots , e_m \rangle$ in *E* to $\mathbb {R}_+$ such that

$$\begin{aligned} \mathbf {t}(\langle e_1, \dots , e_m \rangle ) = \frac{\sum _{i = 1}^m \mathrm {w}(e_i)}{\sum _{i = 1}^m g_i} \end{aligned}$$

when the corresponding edge of $e_i$ in $\mathcal {G}$ is $(u_i, u_{i + 1}, \langle g_i \rangle )$. Also, for all $s,t \in V$, let

$$\begin{aligned} \mathcal {S}_{s,t} := \arg \min \{ \mathbf {t}(P) : P \in \mathcal {P}_{s,t} \} \end{aligned}$$

when $\mathcal {P}_{s,t}$ is a set of all paths from *s* to *t* and $\arg \min$ returns a set of paths such that all members of the set minimize the value of $\mathbf {t}(P)$. From the notation, we have the following lemma.

#### **Lemma 1**

*Let* $s \in V^{(\ell )}$ *and* *e be an edge in* $\mathcal {G}$.* The edge* *e* *is in the set* $S_{(u,\langle i \rangle )}$,* if and only if there is a path* $P \in \mathcal {S}_{s,(u,\langle i \rangle )}$ *such that* $e \in P$.

#### Proof

For any path $\langle e'_1, \dots , e'_{m'} \rangle$ from $s \in V^{(\ell )}$ to $(u,\langle i \rangle )$, if the corresponding edge of $e_i$ in $\mathcal {G}$ is $e'_i = (u'_i, u'_{i + 1}, \langle g'_i \rangle )$, then we have $\sum _{i = 1}^{m'} g'_i = i - \ell$. Thus, a path $P_1$ from $s \in V^{(\ell )}$ to $(u,\langle i \rangle )$ has a smaller summation of weights than a path $P_2$ from $s \in V^{(\ell )}$ to $(u,\langle i \rangle )$, if and only if $\mathbf {t}(P_1) \le \mathbf {t}(P_2)$. $P^*$ is a shortest path from $s \in V^{(\ell )}$ to $(u,\langle i \rangle )$, if and only if $P^*$ minimizes $\mathbf {t}(P)$ and $P^* \in \mathcal {S}_{s,(u,\langle i \rangle )}$. Since $e \in S_{(u,\langle i \rangle )}$ if and only if *e* is in some shortest path from *s* to $(u,\langle i \rangle )$, we can prove this lemma. $\square$

We will use our assumption that the input periodic graph is a weight-transit graph in the following theorem.

#### **Theorem 3**

*If, for all* $e \in \left( (u',\langle i' \rangle ),(u,\langle i \rangle )\right) \in \mathcal {E}$, $i - i' = \mathrm {w}(e)$,* then*

$$\begin{aligned} S_{(u,\langle i \rangle )} = \{ \left( (u',\langle i' \rangle ),(u,\langle i \rangle )\right) \in E : (u',\langle i' \rangle ) \text { is reachable from } s \}. \end{aligned}$$

#### Proof

Based on the assumption, we know that all paths *P* from *s* to $(u, \langle i \rangle )$ has $\mathbf {t}(P) = 1$. Therefore, $\mathcal {S}_{s, (u,\langle i \rangle )}$ is a set of all paths from *s* to $(u,\langle i \rangle )$. The edge $((u',\langle i' \rangle ), (u,\langle i \rangle ))$ is included in one of the paths from *s* to $(u,\langle i \rangle )$, if and only if $(u',\langle i' \rangle )$ is reachable from *s* and there is an edge from $(u',\langle i' \rangle )$ to $(u,\langle i \rangle )$. By Lemma 1, we know that $((u',\langle i' \rangle ), (u,\langle i \rangle )) \in S_{(u,\langle i \rangle )}$, if and only if $\left( (u',\langle i' \rangle ),(u,\langle i \rangle )\right) \in E$ and $(u',\langle i' \rangle )$ is reachable from *s*. $\square$

Our result can be also applied to the case when the input periodic is not a weight-transit periodic graph. Recall that we denote the set of edges in the static graph by $\mathcal {E} = \{e^{(1)}, \dots , e^{(m)} \}$. Let $e^{(i)} := (\nu _i, \mu _i, \langle g_i \rangle )$ and

$$\begin{aligned} \mathcal {E}_M := \left\{ e^{(i)} \in \mathcal {E} : \frac{\mathrm {w}(e_i)}{g_i} = \min \limits _{1\le j \le m} \frac{\mathrm {w}(e_j)}{g_j} \right\} . \end{aligned}$$

If the subgraph of the static graph $(\mathcal {V}, \mathcal {E}_M, \mathrm {w})$ is strongly connected, we can calculate the betweenness centrality using only the edges in $\mathcal {E}_M$. That is because any path that contains an edge in $\mathcal {E} \backslash \mathcal {E}_M$ is not a shortest path. When we know that the shortest paths contain only edges in $\mathcal {E}_M$, we can follow the same argument in Lemma 1 and Theorem 3 to obtain the following result:

#### **Theorem 4**

*If* $(\mathcal {V}, \mathcal {E}_M, \mathrm {w})$ *is strongly connected, and*

$$\begin{aligned} E_M = \{((i, \langle h \rangle ), (j, \langle h \rangle + \langle g \rangle )) : \langle h \rangle \in \mathbb {Z}, ((i, j), \langle g \rangle ) \in \mathcal {E}_M\}, \end{aligned}$$

*Then, we have*

$$\begin{aligned} S_{(u,\langle i \rangle )} = \{ \left( (u',\langle i' \rangle ),(u,\langle i \rangle )\right) \in E_M : (u',\langle i' \rangle ) \text { is reachable from } s \}. \end{aligned}$$

### Example

**Fig. 2:**
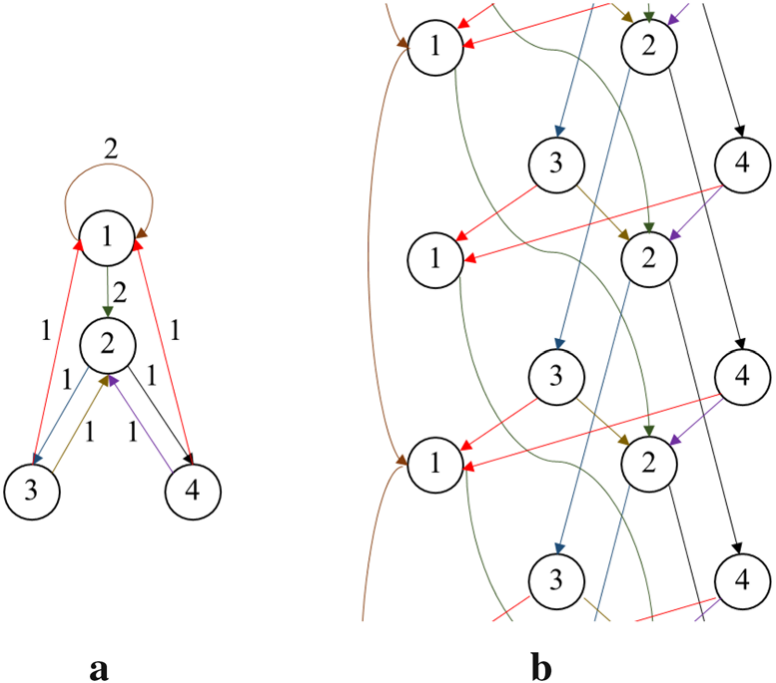
Input graphs for the ‘Example’ subsection **a** static graph **b** periodic graph

In this subsection, we will use a periodic graph shown in Fig. 2 to explain our algorithm given in this section. To simplify our notation, we will denote a vector with only one integer $\langle g \rangle$ by *g* here.

The static graph is $\mathcal {G} = (\mathcal {V}, \mathcal {E}, \mathrm {w})$ where $\mathcal {V} = \{1,2,3,4\}$, and

$$\begin{aligned} \mathcal {E} =\,\left\{ e^{(1)}, \dots , e^{(8)}\right\}= \,& {} \{(1,1,2), (1,2,2, (2,3,1), (2,4,1), (3,1,1), \\&~~~ (3,2,1), (4,1,1), (4,2,1)\} \end{aligned}$$

Let $e^{(i)} = \left( \mu _i, \nu _i, g_i\right)$. We have $\mathrm {w}\left( e^{(i)}\right) = g_i$, because we assume that the graph is a transit-weight periodic graph. The period *p*(*G*) is equal to 2.

In our static graph, all edges are reachable from all nodes. Thus, we get the following recurrence relation for any $s \in G$:

$$\begin{aligned} \sigma _{s,(1,\ell )}= \,& {} \sigma _{s,(1,\ell - 2)} + \sigma _{s,(3,\ell - 1)} + \sigma _{s,(4,\ell - 1)}\\ \sigma _{s,(2,\ell )}= \, & {} \sigma _{s,(1,\ell - 2)} + \sigma _{s,(3,\ell - 1)} + \sigma _{s,(4,\ell - 1)}\\ \sigma _{s,(3,\ell )}=\, & {} \sigma _{s,(2,\ell - 1)}\\ \sigma _{s,(4,\ell )}= \, & {} \sigma _{s,(3,\ell - 1)} \end{aligned}$$

When $s = (1, 0)$, we have $\sigma _{s,(u,\ell )} = 0$ for all $u \in \mathcal {V}$ and $\ell < 0$. For $\ell = 0$, $\sigma _{s,(1,0)} = 1$ and $\sigma _{s,(u,0)} = 0$ if $u \ne 1$. By solving the recurrence relation, for $t > 0$, we have

$$\begin{aligned} \sigma _{(1,-\ell ),(1,0)} = \sigma _{(1,-\ell ),(2,0)} = \sigma _{(1,0), (1,\ell )} = \sigma _{(1,0), (2,\ell )} = {\left\{ \begin{array}{ll} 3^{\ell /2 - 1} & \quad \ell \bmod 2 \equiv 0, \\ 0 & \quad \text {otherwise}, \end{array}\right. } \end{aligned}$$

$$\begin{aligned} \sigma _{(1,-\ell ),(3,0)} = \sigma _{(1,-\ell ),(4,0)} =\sigma _{(1,0), (3,\ell )} = \sigma _{(1,0), (4,\ell )} = {\left\{ \begin{array}{ll} 3^{(\ell - 1)/2 - 1} & \quad \ell \bmod 2 \equiv 1 \text { and } \ell > 1, \\ 0 & \quad \text {otherwise}. \end{array}\right. } \end{aligned}$$

Similarly, we have

$$\begin{aligned} \sigma _{(2,-\ell ), (1,0)} = \sigma _{(2,-\ell ), (2,0)} = \sigma _{(2,0), (1,\ell )} = \sigma _{(2,0), (2,\ell )} = {\left\{ \begin{array}{ll} 2\cdot 3^{\ell /2 - 1} & \quad \ell \bmod 2 \equiv 0, \\ 0 & \quad \text {otherwise}, \end{array}\right. } \end{aligned}$$

$$\begin{aligned} \sigma _{(2,-\ell ), (3,0)} = \sigma _{(2,-\ell ), (4,0)} = \sigma _{(2,0), (3,\ell )} = \sigma _{(2,0), (4,\ell )} = {\left\{ \begin{array}{ll} 2\cdot 3^{(\ell - 1)/2 - 1} & \quad \ell \bmod 2 \equiv 1, \\ 0 & \quad \text {otherwise}. \end{array}\right. } \end{aligned}$$

When $u \in \{3,4\}$, we have

$$\begin{aligned} \sigma _{(u, -\ell ), (1,0)} = \sigma _{(u, -\ell ), (2,0)} = \sigma _{(u, 0), (1,\ell )} = \sigma _{(u, 0), (2,\ell )} = {\left\{ \begin{array}{ll} 3^{(\ell -1)/2} & \quad \ell \bmod 2 \equiv 1, \\ 0 & \quad \text {otherwise}, \end{array}\right. } \end{aligned}$$

$$\begin{aligned} \sigma _{(u, -\ell ), (3,0)} = \sigma _{(u,-\ell ), (4,0)} = \sigma _{(u, 0), (3,\ell )} = \sigma _{(u,0), (4,\ell )} = {\left\{ \begin{array}{ll} 3^{\ell /2 - 1} & \quad \ell \bmod 2 \equiv 0, \\ 0 & \quad \text {otherwise}. \end{array}\right. } \end{aligned}$$

Now, we calculate the betweenness centrality of (1, 0). We know from the solutions of the recurrence relations that a node $(1,\ell ')$ and a node $(1, \ell )$ will have a shortest path that includes (1, 0), if and only if $\ell ', \ell \bmod 2 \equiv 0$. When $\ell ' < 0$ and $\ell > 0$, the number of the shortest paths is

$$\begin{aligned} \sigma _{(1,\ell '),(1,0)} \cdot \sigma _{(1,0),(1,\ell )} = 3^{(\ell - \ell ')/2 - 2}. \end{aligned}$$

Since the number of the shortest paths between $(1,\ell ')$ and $(1,\ell )$ is $3^{(\ell ' - \ell )/2 - 1}$, one-third of the shortest paths passes through (1, 0). By the same argument, we know that, if there is a shortest path from a node $(\nu ', \ell ')$ to a node $(\nu ,\ell )$, that passes through (1, 0), then one-third of the shortest paths between the points passes through (1, 0). Let $S'_D$ be a set of nodes in $V_D$ which has a path to (1, 0), and let $S_D$ be a set of nodes in $V_D$ which has a path from (1, 0). Based on Theorem 3, we know that there is a shortest path from $\left( u', \ell '\right)$ to $(i, \ell )$ that pass through (1, 0), if $\left( u', \ell '\right) \in S_D'$ and $(u, \ell ) \in S_D$.

Since we know that

$$\begin{aligned} \lim \limits _{D \rightarrow \infty }\frac{|S_D|}{|V_D|} = \lim \limits _{D \rightarrow \infty }\frac{|S'_D|}{|V_D|} = \frac{1}{4}, \end{aligned}$$

$$\begin{aligned} pbc((1,0)) = \lim \limits _{D \rightarrow \infty } \left[ \frac{1}{|V_D|^2} \left( \sum \limits _{(\nu ', \ell ')\in S_D', (\nu , \ell ) \in S_D} \frac{1}{3} + \sum \limits _{(\nu ', \ell ') \in S'_D} 1 + \sum \limits _{(\nu , \ell ) \in S_D} 1\right) \right] = \frac{1}{48}. \end{aligned}$$

Using the same argument, we have $pbc((2,0)) = 1/24$, $pbc((3,0)) = 1/32$, and $pbc((4,0)) = 1/32$.

The conventional betweenness centralities of the nodes 1, 2, 3, and 4 of the static graph in Fig. 2a are 6, 10, 6.5, and 6.5, respectively. Although two results look similar, we can observe from the periodic graph that the nodes 3 and 4 are much more important than the node 1 but the classical betweenness centralities of nodes 3 and 4 are larger than that of node 1 by only 8.3 %. By means of our definition and algorithm, the betweenness centralities of nodes 3 and 4 are larger than that of node 1 by 50 %. Therefore, we strongly believe that our betweenness centralities are better than the classical definition for the 1-dimensional periodic graph. In the following section, we provide experimental results to prove the above belief.

## Experimental results

Our experimental settings and results are as follows.

### Dataset

As temporal networks with periodic graphs are an area of emerging research, there are not so many published works of datasets with clustering information. We choose to construct a periodic graph based on a dataset collected for a previous research [21].^2^ In that paper, the authors installed a devise on 125 high school students to detect all of their communications during four school days.

The dataset contains 28,561 communication records. Each record consists of IDs of two students who make a communication, and a time stamp in which that communication occurs. We observe from the dataset that there is a clear periodic pattern in those communication records. All the students communicate with their friends on daily basis (or even on hourly basis with closest ones).

We construct our static graph $\mathcal {G} = (\mathcal {V}, \mathcal {E}, \mathrm {w})$ from that observation. Each node in $\mathcal {V}$ represents a student. An edge $(i, j, \langle g \rangle )$ is in the edge set $\mathcal {E}$, if student *i* communicates with student *j* once in every *g* hours, and the weight of an edge $(i, j, \langle g \rangle )$ is equal to *g*. As a result of this construction, we get a static graph with 125 nodes and 455 edges.

Based on the static graph, we will get a periodic graph $G = (V, E, \hat{\mathrm {w}})$, where $(i, \langle h \rangle ) \in V$ represents a student *i* at time *h*. An edge $\left( (i, \langle h \rangle ), (j, \langle h + g \rangle )\right) \in E$ represents the fact that the information known by *i* at time *h* will be known by *j* at time $h + g$, as *i* talks with *j* once in every *g* hours. This is because the high school students have a fixed class schedule and they only share physical location with people for other classes (and can speak freely) in very specific situations such as lunch breaks or between-class breaks.

### Computational time

We implement our betweenness centrality algorithm and the fastest algorithm for finite graph in [20] using Python, and run the program on a personal computer with Intel(R) Core(TM) i7-3770 @ 3.40GHz CPU, Windows 8.1 64 bits, 16GB RAM. Our algorithm takes only 3.2 s for the periodic graph constructed in the previous subsection, while the previous algorithm takes 0.4 seconds for computing betweenness for the static graph, resulting in only an eightfold slower computation time compared to that when computing it on the infinite periodic graph.

**Fig. 3:**
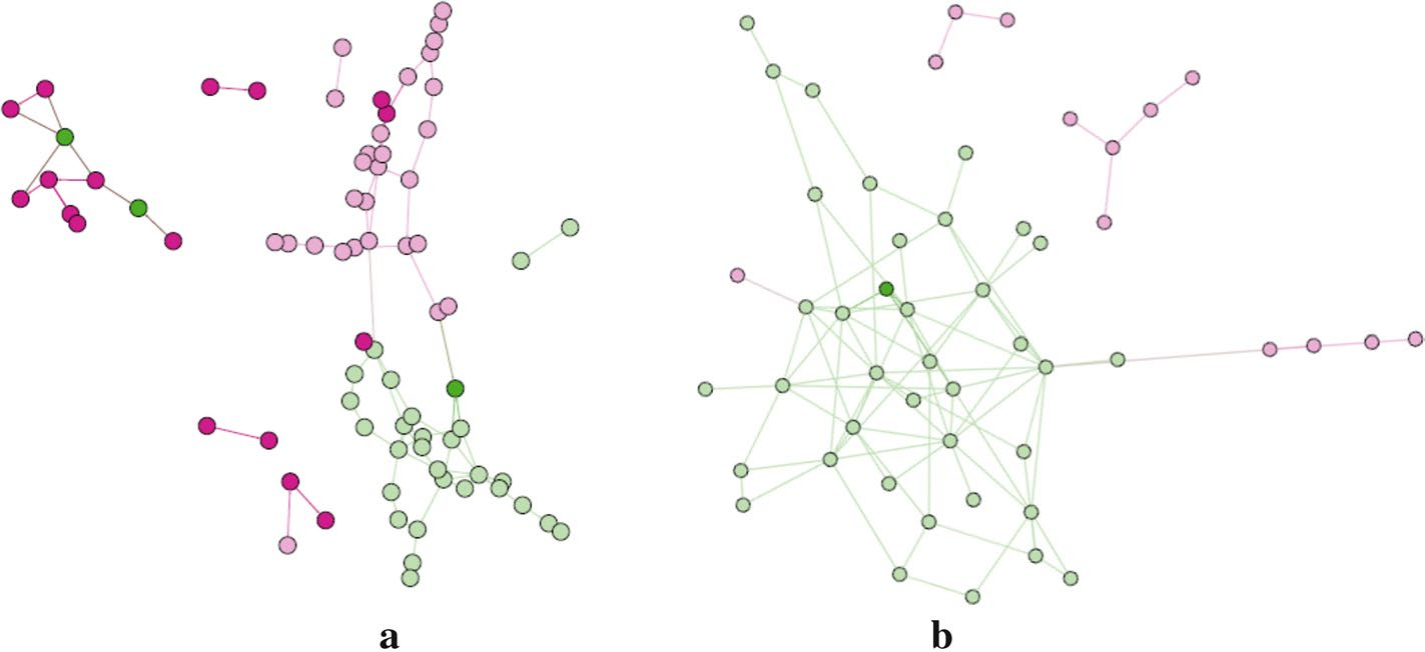
Edge-betweenness clusters using betweenness values on static graph (*left*) and periodic graph (*right*); Each *node* represents a student, and the *color* of each node represents the class of a student corresponding to the node. The edges shown in the figures are edges that remained after we remove edges with high betweenness centrality. Each connected component in the remaining graphs represents a cluster of nodes

### Clustering using *pbc*(*v*)

We can also find each edge-betweenness using the edge-partition technique, and thereby the betweenness of that middle node will be the edge betweenness. One of the most common clustering methods is to remove edges with highest betweenness, and group nodes that are in the same connected component into a cluster.

In this experiment, we set the number of removed edges to $p \times 455$ when *p* is a real number between 0.1 and 1.

**Fig. 4:**
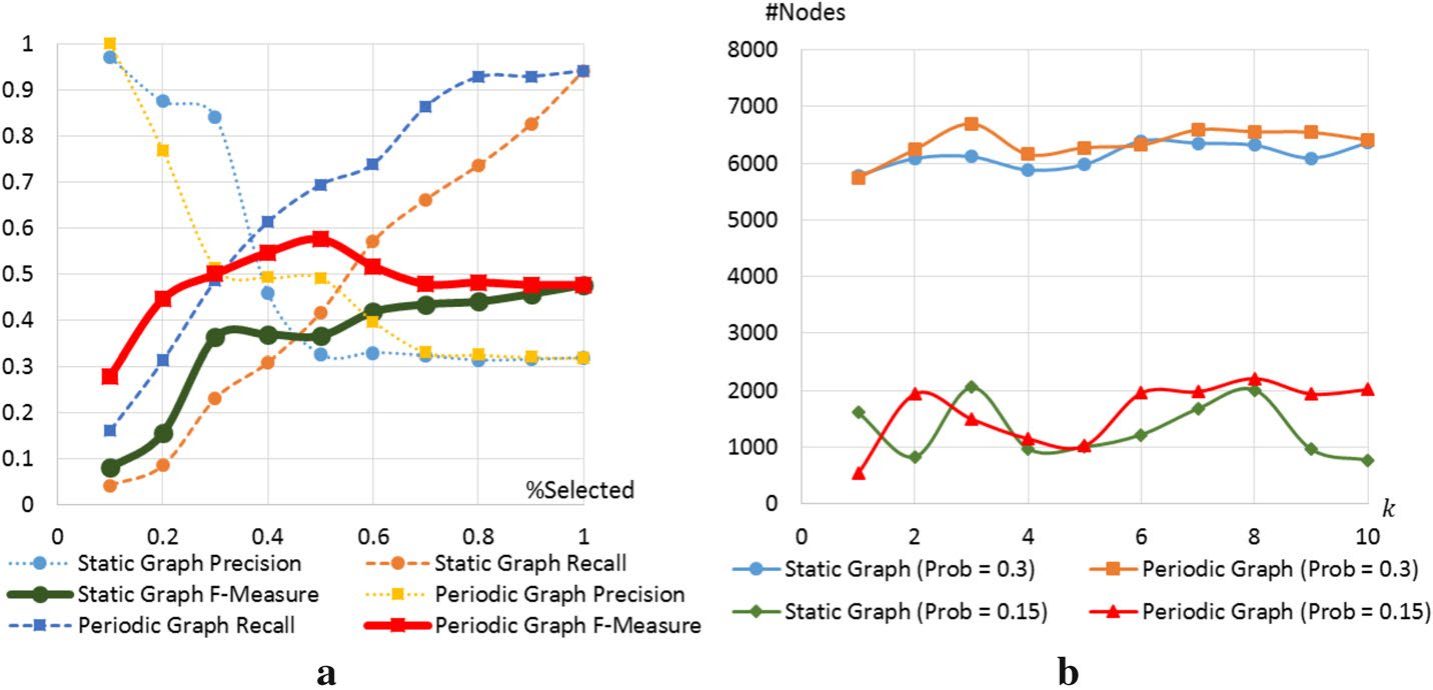
Comparison between our results and previous works. **a** clustering results. **b** influence maximization

In Fig. 3, we compare the clustering results obtained by removing edges with high infinite betweenness and the results obtained by removing edges with high betweenness in static graph when $p = 0.5$. The color of each node represents a class of each student given in our dataset. Two nodes are considered to be in the same cluster, if they are connected in the result graphs. We can clearly see from the figure that the pink nodes and the green nodes are put into the same cluster in the conventional clustering results, while all clusters are almost unicolor in our clustering results.

In Fig. 4, clustering results are evaluated by the precision, the value, and the F-measure calculated from the results and clusters given in the dataset. Although our precision is smaller than the value from the previous method in some *p*, our recall is significantly larger for all *p*. Therefore, our F-measure is also larger for all *p*. When $p = 0.5$, we improve the precision by 51 %, recall by 66 %, and F-measure by 57 %.

### Maximizing influence using *pbc*(*v*)

In this subsection, we intend to model the way some information spreads over the students (e.g., a rumor). For this purpose, we select *k* students, with *k* being an integer between 1–10, and with probability $p \in \{0.15, 0.3\}$, the selected students will send information to node adjacent to them in $\mathcal {G}$. The nodes who receive the information will forward the information with the same probability after adding more content to it.

Because more contents are added, students who did forward the information may forward the message again. To assure that a large number of students can get several contents added during the process, we intend to maximize the number of nodes that are forwarded information in periodic graph.

In Fig. 4b, it can be clearly seen that nodes selected by periodic betweenness centrality can affect more nodes than nodes selected by betweenness centrality in static graph. As seen from our results, it can affect up to 20 % more nodes than the conventional method when $k = 2$ and $p = 0.15$, and up to 9.9 % when $k = 8$ and $p = 0.3$.

### Synthesized dataset

To confirm that our algorithm is scalable, we perform an experiment on datasets synthesized from a Facebook ego network. The network has 4039 nodes and 88234 edges, and can be obtained at the Stanford large network dataset collection (SNAP) [44].

Our datasets are subgraphs of the Facebook ego network. The numbers of nodes in each subgraph are 200, 300, 400, 500, 600, 700, 800, 900. To find the subgraphs, we begin from a node with highest degree, and perform a breadth-first search from the node. We stop the search when the number of nodes reaches the desired number. Our subgraphs are subgraphs induced by the set of nodes found by the search. The numbers of edges in the subgraphs with 200, 300, 400, 500, 600, 700, 800, and 900 are 962, 2046, 3120, 3513, 4326, 5635, 7448, and 9976, respectively. The weight of the edges are chosen randomly from the set $\{1,2,3,4,5,6,7,8,9,10,11,12\}$.

Although there are many previous works that generated the subgraphs by picking nodes at random (e.g., [45]), the method does not work in this experiment as it outputs sparse graphs with a large number of small connected components. We strongly believe that the subgraphs obtained from the breadth-first search algorithm can maintain properties of the social network such as small-world phenomenon.

**Fig. 5:**
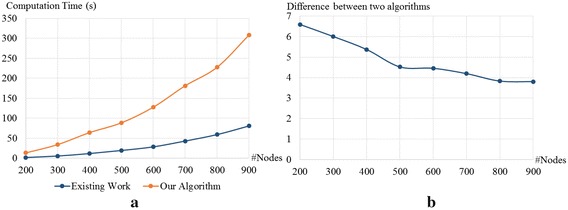
Computation times when the inputs are synthesized from Facebook dataset obtained from [44]. **a** Comparison between the computation times for the periodic betweenness centrality and the classical centrality on the static graphs. **b** The computation times for the periodic betweenness centrality divided by the computation time for the classical centrality on the static graphs

In Fig. 5a, for the subgraphs of the Facebook ego network, we compare the computation time of our method to those of the method calculating the betweenness centralities of static graphs. When the number of nodes becomes larger, the difference between the computation times becomes larger. However, when we divide the computation time for the static graphs with our computation result, the division results become smaller when the number of nodes becomes larger. We can see from Fig. 5b that, when the number of nodes is 200, our computation time is about 6.6 times of that in the static graphs. When the number of nodes is 900, our computation time is only about 3.8 times of that of the previous works. From these results, we expect that our computation time is not that larger than the time in the previous works in a large graph.

**Fig. 6:**
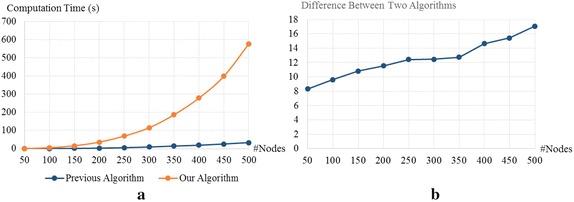
Computation times when the inputs are random graphs generated from Erdős-Rényi model [46]. **a** Comparison between the computation times for the periodic betweenness centrality and the classical centrality on the static graphs. **b** the computation times for the periodic betweenness centrality divided by the computation time for the classical centrality on the static graphs

We also compare the computation times of our algorithm and the algorithm for static graphs when the input networks are random graphs. We generated the graphs using the Erdős-Rényi model. The numbers of nodes in the graphs are 50, 100, 150, 200, 250, 300, 350, 400, and 450. Two nodes in the graphs are connected with probability 0.3. Thus, the numbers of edges in the graph with 50, 100, 150, 200, 250, 300, 350, 400, and 450 are about 377, 1485, 3353, 5970, 9338, 13455, 18323, 23940, 30308, and 37425, respectively. The computation times for this dataset are shown in Fig. 6a. Unlike the computation times in Fig. 5a, we found that the difference between our computation time and the previous computation time becomes larger when the number of nodes is larger.

**Fig. 7:**
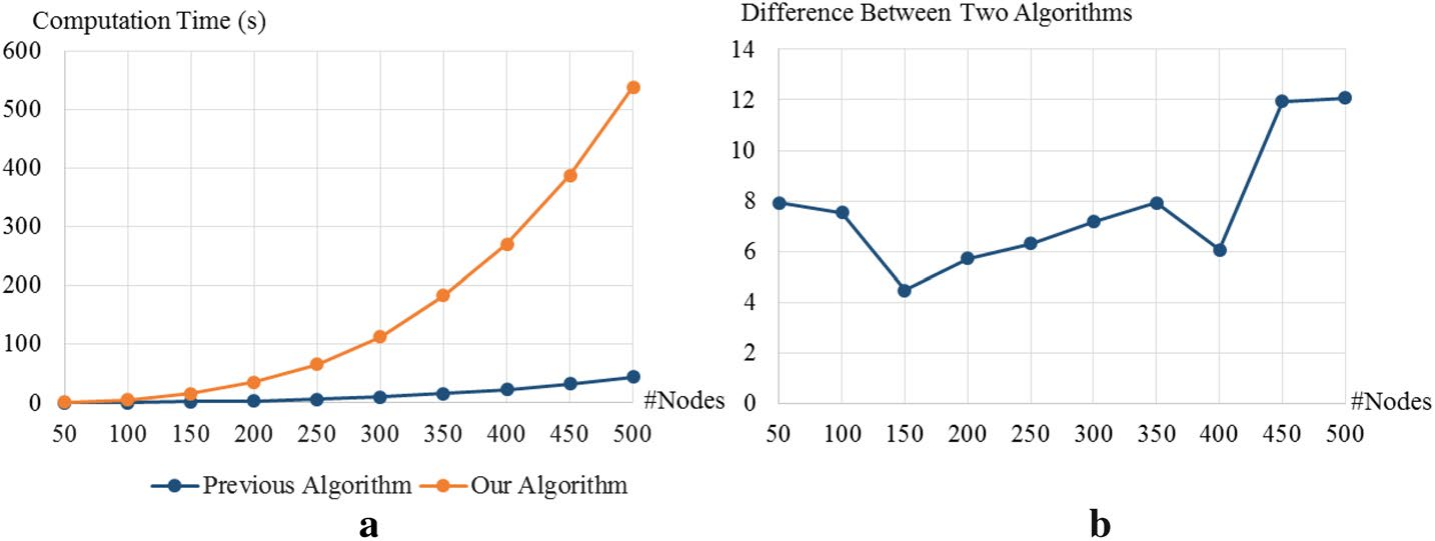
Computation times when the inputs are graphs with small-world properties generated from Watts-Strogatz model [47]. **a** Comparison between the computation times for the periodic betweenness centrality and the classical centrality on the static graphs. **b** The computation times for the periodic betweenness centrality divided by the computation times for the classical centrality on the static graphs

Next, we discuss our experimental results when the input networks have small-world properties. To generate graphs that have such properties, we use the Watts-Strogatz model [47]. Similar to the random graphs, the numbers of nodes in the graphs are 50, 100, 150, 200, 250, 300, 350, 400, and 450, and two nodes in the graphs are connected with probability 0.3. In this setting, the difference between two computation times does not clearly increase when the number of nodes increases. However, we can still observe the increasing trend in Fig. 7b.

The results obtained from the subgraphs of the Facebook ego network are different from those obtained from random graphs and graphs with small-world properties. We believe that the reason behind that is the number of edges in each input graph. The number of edges in graphs obtained from the ego network grows linearly with the number of nodes, while the number of edges in the other two datasets grows quadratically. We know from the results that the difference between our computation time and the previous computation time tends to be larger when the number of edges is larger. That is surprising, since our computational complexity given in Theorem 2, $O(|\mathcal {V}|^3)$, does not depend on the number of edges, while the complexity of the previous algorithm, $O(|\mathcal {V}||\mathcal {E}|)$, depends on the number of edges. Based on this result, we strongly believe that our analysis can be improved to reduce our computational complexity to the other level depending on the number of edges.

Although our computation time is not much larger than that of the previous works, our memory consumption is much larger than them. While the previous algorithms use only $O(|\mathcal {V}|)$ memory, we use $O(|\mathcal {V}|^2 p^2(G))$. Because, in these synthesized datasets, the value of *p*(*G*) can be as large as 27,720, we cannot perform an experiment on the datasets with more than 1000 nodes. Reducing the memory consumption in our algorithm is currently one of the goals that we are aiming for.

## Conclusion and future work

It usually takes long computation time to extract information from a temporal network, as the number of nodes in the graph is usually exceptionally large. We can reduce that computation time if the network can be specified as a repetitive structure of a small graph, called the static graph. In this paper, we propose an efficient algorithm that can compute betweenness centrality of that infinite network. The computation time of the algorithm proposed is comparable to the time that the fastest method required for the static graph.

Currently, we are aiming to find more applications of the betweenness centrality on the periodic graph, other than the clustering and the influence maximization. Also, we are planning to collect information to construct more periodic datasets, and use those datasets to show that our results are more preferable than the results obtained when using previous methods on static graph. Besides, we plan to find a mathematical model that can capture properties of opportunistic networks. We will use the model to generate a large periodic graph, before using that large graph to test if our algorithm is scalable enough in those practical settings.

Although the time to exactly calculate the betweenness centrality is as large as $O(|\mathcal {V}||\mathcal {E}|)$, there are scalable algorithms proposed to approximate the value of the centrality (e.g., [29]). In future work, we aim to devise an algorithm for approximating the periodic betweenness centrality that can terminate in $O(|\mathcal {V}|)$.

## Footnotes

1 This manuscript is an extension of our paper previously published in the proceeding of CSoNet’15 [1].

2 The dataset is published at http://www.sociopatterns.org.
